# Physical Properties and Volatile Profile Changes of Cauliflower Treated with Onion and Beetroot Juices Using Vacuum Impregnation Process

**DOI:** 10.3390/molecules30102147

**Published:** 2025-05-13

**Authors:** Magdalena Kręcisz, Bogdan Stępień, Marta Klemens, Aleks Latański

**Affiliations:** 1 Institute of Agricultural Engineering, Wrocław University of Environmental and Life Sciences, Chełmońskiego Street 37a, 51-630 Wrocław, Poland; bogdan.stepien@upwr.edu.pl (B.S.); 120609@student.upwr.edu.pl (A.L.); 2 Department of Fruit, Vegetable and Grain Technology, Wroclaw University of Environmental and Life Sciences, Chełmońskiego Street 37/41, 51-630 Wrocław, Poland; marta.klemens@upwr.edu.pl

**Keywords:** cauliflower, beetroot, onion, vacuum impregnation, volatile compounds, drying, bulk density, texture

## Abstract

The use of vacuum impregnation with onion and beetroot juice can help with the challenge of modifying plant tissue and fits in with current trends in the development of plant-based snacks. This study aimed to determine the effect of vacuum impregnation (VI) as a pretreatment before drying on the volatile compounds, texture profile, color, dry matter, water activity, and density of cauliflower. The pretreatment was carried out at a pressure of 0.06 MPa, and the total process time was 21 min. Two types of impregnation solutions were used: onion juice and beetroot juice. The samples were dried by freeze-drying and vacuum. Numerous volatile compounds were identified: twenty-two for raw cauliflower, twenty-nine for cauliflower after VI with beetroot juice, and twenty-four for cauliflower after vacuum impregnation with onion juice. The following volatile compounds were present in the highest amounts: 1-heptene, 2-methyl-(>60%), 2-ethylcyclobutanol (>4%), nona-3,5-dien-2-one (>1.8%), and two unidentified compounds, unknown 1 (probably an isomer of nona-3,5-dien-2-one (>1.8%)) and unknown 2 (probably a fatty acid) (>9.8%). The pretreatment had a significant effect on water activity, density, texture profile and color. The freeze-drying method proved to be effective in obtaining lower values of water activity and density. In addition, dried products obtained by this method were characterized by a higher degree of color recovery after rehydration and textural properties similar to fresh raw material. The use of different impregnation solutions had a significant impact on the properties studied. The greatest color change occurred in cauliflower treated with beetroot juice.

## 1. Introduction

Dried vegetable snacks can be a healthy alternative for people who care about a healthy lifestyle [1] or suffer from celiac disease or food intolerance. People with food intolerances and celiac disease must follow a special diet. This is now a growing health problem related to gluten sensitivity and absorption disorders [2].

Cauliflower (*Brassica oleracea var. botrytis*), belonging to the cabbage family (*Brassicaceae*), is one of the most important vegetable crops in the world. It is a plant with an edible head, called a “rosette”, consisting of tightly packed flower buds, usually white, but sometimes purple or orange. It originates from the Mediterranean region and its cultivation became widespread in the 15th century. Cauliflower can be eaten raw, boiled, or baked [3]. It is characterized by its high nutritional value and low calorie content, which makes it a valuable ingredient in the diet. It contains vitamins such as C, K, A, and B6. In addition, it is a rich source of fiber and bioactive compounds, including glucosinolates, which may have anticancer properties [4,5]. Cauliflower is rich in nutrients, including B vitamins, especially niacin. It contains nicotinic and pantothenic acids as well as minerals such as calcium, magnesium, potassium, iron and selenium. It is also a source of antioxidants such as flavonoids, polyphenols, folic acid, and carotene [6,7].

Drying is a process used to extend the shelf life of food by removing moisture and thus inhibiting the growth of microorganisms [8,9,10,11]. Drying is a well-known process in the food industry, thanks to which water activity is reduced to a level as low as 0.8. This level of water activity prevents the growth of most bacteria, which is why dried food products are considered safe and can be stored for longer [9].

Out of the many drying processes used for food preservation, freeze-drying (FD) is considered to be the method that best preserves the quality indicators of food, i.e., size, appearance, shape, color, taste, and aroma. Dry materials obtained by the freeze-drying method have the ability to rehydrate and are brittle, highly porous, and hygroscopic [9,10]. This method, thanks to the effect of low temperatures and a strong vacuum, is especially recommended for materials sensitive to high temperatures [12]. Vacuum drying (VD) is carried out under reduced pressures, generally not higher than 30 kPa [12]. The food market is constantly being driven by, among others, different drying techniques. Consumers are interested in new health-promoting products; therefore, it is essential to produce dried products with the right visual and olfactory characteristics [13].

Additionally, pretreatment methods can be used before the drying process to modify physicochemical properties, volatile compounds, or texture [13]. Vacuum impregnation as a pretreatment has been used in various material matrices, e.g., broccoli [13], zucchini [13,14], sweet potato [15], celery [16], fish [17], meat [18], and cheese [19]. Under vacuum conditions, gases in the pores and intercellular spaces of cellular porous materials expand to reach a state of thermodynamic equilibrium with the environment. This process partially removes the gas phase from the product matrix (degassing process). When the material is immersed in a liquid impregnation solution, capillary action facilitates the penetration of the liquid into the pore system [18,19,20]. The degree of solution infiltration is a function of structural parameters such as porosity, degree of interconnectivity, and pore diameter. The restoration of atmospheric pressure generates a macroscopic pressure gradient between the chamber volume and the internal porous architecture, which enhances the transport of liquid into the material. The final volume of the adsorbed solution is determined by the effectiveness of the degassing process during the vacuum application phase [19]. Pretreatment affects the kinetics of the drying process (shortening the drying time) [16], color, total phenol content [15], and antioxidant activity [15] and prompts a change in bioactive compounds [14]. The studies presented by Derossi et al. [21] showed that the use of the vacuum impregnation process improved Ph reduction in carrot and eggplant slices compared to traditional acidification–immersion. VI enables a rapid change in the structure and composition of the product [22]. In studies on celery [23] and zucchini [14], a change in chemical composition was observed after a vacuum impregnation process using vegetable impregnation solutions. Studies by Demir et al. [18] using VI to marinate beef in onion juice showed that the impregnation solution used reduced lipid oxidation and increased the overall flavor score in a sensory evaluation [18].

Even though there are studies on vacuum impregnation in our bibliography, there are no reports on cauliflower. Our other studies confirmed the positive effect of onion and beetroot juice on the physicochemical properties of plant tissue, which is why we focused on these juices [15,16]. Onion juice has a high content of bioactive compounds that have various health benefits, such as anti-obesity, anti-diabetic, and antioxidant properties [24]. Furthermore, onions have a rich profile of volatile compounds [25], and beetroot has an intense burgundy color [16]. The study of the vacuum impregnation of cauliflower using beetroot and onion juices is of great importance due to its novelty and potential health and sensory benefits. Despite the growing interest in vacuum impregnation technology, scientific reports to date do not include white cauliflower as a raw material or the use of natural plant juices with high bioactive value. Onion juice provides valuable biologically active compounds with antioxidant, anti-diabetic, and anti-obesity effects, as well as volatile aromatic compounds, while beetroot juice is a rich source of natural dyes. The use of these juices not only enriches the nutritional value of the product, but can also positively affect its color, aroma, and sensory acceptability, which makes this method promising from the perspective of the food industry and functional foods.

Thus, in the study that is the subject of this paper, vacuum impregnation was used as a pretreatment prior to the drying process of cauliflower to investigate the effect of onion and beetroot juice on its volatile compound content, texture profile, color, density, water activity, and dry matter. Knowledge of these physicochemical properties can help to optimize the processing and design of cauliflower dehydration, which can be an innovative product or food additive.

## 2. Results and Discussion

### 2.1. Volatile Organic Compound (VOC) Profiles

The full VOC profiles of the samples are shown in Table 1 and Table 2. Twenty-two VOCs were found in the cauliflower, while twenty-nine VOCs were present in the profile of the cauliflower with beetroot juice, and twenty-four VOCs in the profile of the cauliflower with onion juice. After the drying process, the number of identified compounds in the cauliflower increased to twenty-seven, in the cauliflower impregnated with beetroot juice to thirty, and with onion juice to thirty-one.

In the case of fresh cauliflower, impregnated both with beetroot and onion juice as well as the pure vegetable, the highest amount of volatile compounds consisted of the following: 1-heptene, 2-methyl-(>60%), 2-ethylcyclobutanol (>4%), nona-3,5-dien-2-one (>1.8%), and two unidentified compounds, unknown 1 (probably isomer of nona-3,5-dien-2-one (>1.8%)) and unknown 2 (probably fatty acid) (>9.8%).

Our previous studies have shown that the impact of vegetable juice impregnation on the profile of volatile organic compounds (VOCs) is highly dependent on the type of plant material. In the case of celery, the use of beetroot juice had only a slight effect on the composition of volatile compounds compared to fresh, non-impregnated celery [16]. In contrast, the use of onion, kale, and celery stalk juices caused significant changes in the aroma profile [23], markedly increasing the proportion of sulfur compounds and green notes. Similar patterns were observed for other vegetables, such as cauliflower, broccoli, and zucchini. Cauliflower exhibited a strong integration of compounds from the impregnation solutions, which became even more intensified after the drying process.

**Figure 1:**
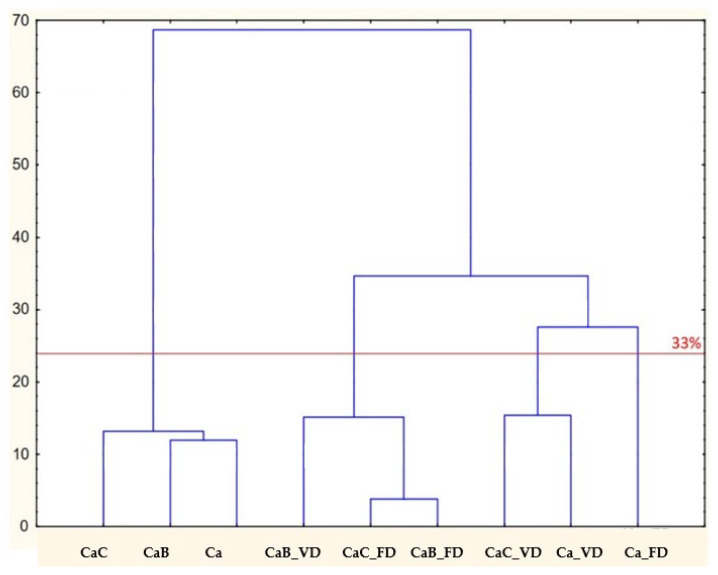
HCA results analysis of fresh and dried cauliflower with beetroot and onion juice. Ca: cauliflower; CaC: cauliflower with onion juice; CaB: cauliflower with beetroot juice; FD: freeze-drying; VD: vacuum drying.

Impregnation with beetroot or onion juice introduces additional volatile compounds into the cauliflower profile, but the drying process significantly modifies this profile (Figure 1). Among the compounds that increase in intensity in dried cauliflower impregnated with juices, myrcene (22–62%) is found at higher levels compared to fresh cauliflower (13–15%). This indicates the presence of these compounds in the impregnating solutions used and the effective impact of the pretreatment on the transport of juice to vegetable tissue. On the other hand, 2-ethylcyclobutanol, present in both fresh and dried products, shows a higher concentration in dried pure cauliflower. A notable intensity, regardless of the drying and impregnation methods, is also shown by an unidentified compound, which is likely a fatty acid (6–22%). In contrast, 1-heptene, 2-methyl-(61–72%), the dominant compound in fresh cauliflower, is practically undetectable in dried products. Impregnation with beetroot juice has a significant impact on the profile of volatile aromatic compounds (VOCs) in cauliflower. A similar effect was observed in studies involving the impregnation of broccoli and zucchini [13]. However, these effects are strongly dependent on the type of vegetable, its structure, and the applied drying process. In the case of fresh cauliflower, impregnation with beetroot juice introduces eight new compounds, such as mentha-1(7),8-diene <meta-> (0.71%), 2-Pentanethiol, 4-methyl-(1.11%), and pentyl furan <2-> (0.24%). This results in the addition of minty, slightly caramel-like, and mildly sulfurous aromas, which refresh and slightly sweeten the natural cabbage-like aroma of cauliflower [26]. Meanwhile, impregnation with onion juice adds compounds such as dimethyl trisulfide (0.67%), hexanol <2-ethyl-> (0.24%), 2-methyl 6-methylene 2-octene (0.62%), and an unidentified compound with an RI of 781 (11.33%). These changes introduce intense sulfurous, green, and vegetable-like notes [27], making the aroma heavier and sharper compared to the fresh, untreated cauliflower [28].

In dried cauliflower, impregnation with beetroot and onion juice leads to the appearance of compounds such as isovaleric acid (0.58–5.22%), pinene <alpha-> (0.38–0.84%) and <beta-> (0.14–0.39%), hept-5-en-2-one <6-methyl-> (1.47–2.15%), decane (0.10–0.69%), mentha-1(7),8-diene <p-> (1.21–3.29%), cymene <para-> (0.71–1.81%), limonene (0.27–0.39%), geranial (0.06–0.19%), and citral (0.03–0.19%). Additionally, the compound isothiocyanate <2-propenyl-> (0.42%) appears only in beetroot-impregnated cauliflower after freeze-drying. As a result, dried cauliflower impregnated with beetroot juice gains fresh minty, citrus, and resinous notes, along with a distinctive pungency reminiscent of horseradish (due to the appearance of isothiocyanate <2-propenyl-> at 0.42%). In contrast, onion juice impregnation after drying produces a more resinous, citrusy aroma with a milder sulfurous background compared to the strongly cabbage-like and earthy profile of untreated dried cauliflower.

In the case of fresh broccoli, impregnation enriched the aroma profile with com-pounds characteristic of green and fruity notes, such as 2-(E)-hexen-1-ol, 2-(Z)-hexen-1-ol, and acetophenone. However, after drying, the differences between the impregnated and non-impregnated broccoli samples became blurred, indicating that the drying technology, rather than the impregnation itself, had the dominant influence on the final VOC profile. Courgette showed a different trend. In its raw state, both the natural and impregnated samples exhibited very similar VOC profiles. Differences only emerged after drying, suggesting that the drying process, rather than impregnation, had the greatest impact on the final volatile compound composition. These results may suggest that compounds from the impregnation are either preserved or modified during thermal processing [13].

Impregnation with onion juice significantly alters the volatile compound profile in vegetables. Before impregnation, the cauliflower profile was dominated by mild, vegetable-like sulfur compounds and aldehydes. After impregnation, there was an increase in strong sulfur compounds, such as dimethyl trisulfide and mercaptans, giving the product a more intense onion-like and sulfurous aroma. The proportion of aldehydes and ketones responsible for green and fresh notes also changed [23]. Raw celery is characterized by a fresh, green-herbal aroma. The primary compound responsible for this profile is myrcene, which imparts a terpene-like, herbal, and slightly rosy scent reminiscent of celery and carrot. The presence of 2-ethylcyclobutanol introduces subtle earthy and woody notes, while 2-methyl-1-heptene may contribute fatty, waxy undertones, enhancing the complexity of the aroma. Benzaldehyde, known for its almond-like odor, adds warm, sweetish tones in small amounts [29]. Nona-3,5-dien-2-one is a key compound providing celery with its distinctive vegetable-like aroma. Butenolide (γ-butyrolactone) intensifies spicy, root-like notes, while tetradecane brings a fatty, waxy background that supports the overall aroma structure. Pentyl formate, in trace amounts, may round out the aromatic profile by adding fruity nuances [30]. The drying process leads to changes in celery’s aromatic profile. Low-boiling-point compounds like myrcene may be reduced, weakening the fresh, green notes. At the same time, the concentration of higher-molecular-weight compounds such as tetradecane and butenolide may increase, giving the product more fatty, waxy, and spicy characteristics. Benzaldehyde may also become more concentrated, enhancing sweet, almond-like tones [31].

In summary, the effects of beetroot juice impregnation are clearly differentiated de-pending on the type of vegetable. Cauliflower exhibits a strong integration of aromatic compounds from the impregnation solution, which persist even after drying. Similarly, onion juice impregnation significantly modifies the aroma profile, particularly by intensifying sulfurous and green notes, although the extent of these changes also depends on the vegetable’s structure and the drying method. In contrast, broccoli and zucchini respond more weakly, with their final VOC profiles being more strongly shaped by the drying technique. This suggests that the vegetable’s structure and chemical composition may influence the transfer efficiency and stability of the aromatic compounds derived from impregnation.

The drying process of impregnated cauliflower has a greater impact on the volatile compound profile than the impregnation of fresh material itself, as evidenced by changes in the intensity and presence of certain volatile compounds. It can therefore be argued that the drying process intensifies the aroma of cauliflower, while impregnation with juices adds complexity to it (Figure 1). Based on the HCA (Figure 1), it can also be concluded that, aside from the differences between the dried and fresh samples, only the freeze-dried material without impregnation exhibits a distinctly different aroma profile.

Based on the cluster analysis, it can be concluded that, at a similarity level of 33%, the studied samples group into four clusters. The first cluster is formed by the samples CaC, CaB, and CaO. These three samples are the most similar to each other and exhibit a comparable profile of volatile compounds. All three samples were not subjected to any drying processes. Despite their impregnation with beetroot and onion juice, their profiles of volatile compounds did not change significantly. The second cluster includes the samples CaB_VD, CaC_FD, and CaB_FD. They are grouped together, indicating similarity likely resulting from the use of drying processes such as vacuum drying (VD) and freeze-drying (FD). These processes introduced changes to the samples, but the changes were similar enough for the samples to be grouped together. It can be concluded that impregnation with onion juice followed by freeze-drying alters the volatile compound profile in a similar way to impregnation with beetroot juice, regardless of the drying method. The third cluster consists of the samples CaC_VD and CaO_VD. Although they originate from different materials (CaC and CaO), the vacuum drying (VD) process caused their volatile compound profiles to become similar, leading to their joint grouping. It can be stated that impregnation with onion juice in the case of vacuum drying does not significantly alter the volatile compound profile. The fourth cluster consists of a single sample, CaO FD. Its distinctness from all other samples indicates that the freeze-drying (FD) process and the lack of impregnation affected it in a unique way, significantly differentiating its VOC profile from the other samples.

In summary, the dendrogram results show that both the type of sample and the technological treatment applied have a crucial impact on the samples’ similarity. Samples without treatment remain very similar to each other, whereas drying processes (vacuum drying and freeze-drying) modify the characteristics of the samples to varying degrees, leading to noticeable differences.

### 2.2. Vacuum Impregnation

The weight gain (WG) and Brix degrees (°Bx) are shown in Table 3. In this study, which used red beetroot juice, the weight gain was 8.90% at 7.5 °Bx compared to cauliflower before vacuum impregnation. However, when onion juice was used, the weight gain was 4.03% at 11.5 °Bx. This indicates the porous structure of the tested material.

### 2.3. Dry Matter (DM), Water Activity (AW), and Density

The dry matter content of the fresh cauliflower was 8.91%, which generally corresponded to the data in the literature (Table 4). The values obtained were slightly higher than those obtained by Kurek et al. (6.9%) [32], Florkiewicz et al. (8.42%) [33], and Cebula et al. (6.57%) [34]. A dry matter value of 9.18% was recorded in purple cauliflower [35]. Kapusta-Duch et al. [35] showed an increase in dry matter content in cauliflower stored frozen (in zip bags) for 2 months of 11.60%. Other authors observed a DM increase of 14.5% for the Romanesco variety, and increases of 22.3% and 10.5% for the white variety [34,35]. At low temperatures, the cell walls denature, which is associated with the release of water, which is why higher dry matter values are observed in materials stored in the freezer [35]. After the vacuum impregnation process, a higher dry matter content was recorded in cauliflower, amounting to 9.22% for CaC and 9.88% for CaB. Similar increases were also recorded for cucumber (from 7.1% to 7.5%) and broccoli (from 15.1% to 15.4%) after VI with beetroot juice [13], celeriac vacuum-packed in beetroot juice (from 9.89 to 10.43%) [16], zucchini in tomato juice (from 5.3 to 7.5%) [36], and sweet potato vacuum-packed in onion juice (from 5.3 to 7.5%) carrot juice (from 5.3 to 7.5%) [15]. Of the tested methods of dehydrating vegetables, higher dry matter values were recorded for all tested materials after the freeze-drying process.

The water activity of the fresh cauliflower was 0.970, the cauliflower after VI with onion juice showed 0.978, and the cauliflower after VI with beetroot juice showed 0.982. The results obtained in this study are consistent with the results obtained for celery after VI with beetroot juice [16] and sweet potatoes after VI with kale juice [15]. The use of drying processes allowed for a satisfactory water activity value to be obtained. The lowest AW was obtained for Ca_FD (0.145). In general, the use of the vacuum impregnation process increased the AW value of both the fresh and dried materials. Of the drying methods used, the lowest values of the tested parameters were recorded for the freeze-dried products (Table 4).

The density of the fresh cauliflower was 275.44 kg/m^3^, while that of the cauliflower after VI with onion juice was 365.94 kg/m^3^, and that after VI with beetroot juice was 368.23 kg/m^3^ (Table 4). The conducted research shows that the use of the vacuum impregnation process increases the density of fresh and dried materials. These results are consistent with the studies conducted for broccoli and zucchini [13]. The density of the dried samples obtained by the freeze-drying method was lower, on average by 76.57%, compared to the vacuum-dried cauliflower. Other researchers have also noted the lowest density values of freeze-dried materials compared to other methods [37].

### 2.4. Color

**Figure 2:**
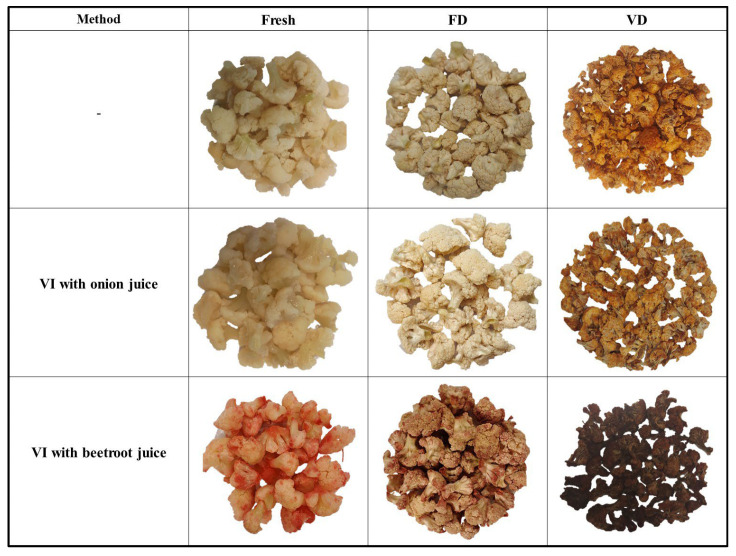
Visual comparison of selected cauliflower pieces subjected to vacuum impregnation (VI) using onion and beetroot juice. FD—freeze-drying; VD—vacuum drying.

Table 5 shows the color parameters of the fresh, impregnated, and dried cauliflower samples. The color profile of the fresh cauliflower was as follows: L*: 73.10; a*: −0.65; b: 11.53. Research presented by Pasten et al. [38] confirms the obtained test results. The authors recorded the following fresh cauliflower profile: L: 70.48; A: −3.68; B: 19.74. Our L* values were higher after drying than for fresh, unprepared cauliflower, which is consistent with the results of the study by Pasten et al. [38]. The highest lightness values (L*) were recorded for freeze-drying, which is in line with studies on celery [16], zucchini [14], cauliflower [38], melon [9], and green beans [8]. And the darkest values were recorded for the cauliflower after vacuum impregnation with beetroot juice and vacuum drying. In addition, the CaC_VD and CaB_VD samples are characterized by a greater loss of brightness and the greatest color difference compared to cauliflower after FD. This may be related to the higher temperature of the vacuum drying process. In the case of fresh cauliflower and after VI with onion juice as well as after FD, the color values and color difference were similar. The greatest differences in brightness were observed for cauliflower impregnated with beetroot juice. This is an expected result due to the dark color of the juice. These results are similar to those obtained for celery [16], in which researchers observed increased color differences from 132% to 523% depending on the drying method. In turn, in studies of zucchini and broccoli in which beetroot juice was used, an increased color difference was observed by an average of 1036% for zucchini and 27% for broccoli [13]. The value of the a* parameter ranged from −0.87 for CaC to 15.67 for CaB. Positive a* values indicate a red color, which is consistent with the visual assessment shown in Figure 2. Positive b* values indicate a yellow color. The strongest yellow color was observed in fresh cauliflower and in cauliflower vacuum impregnated with onion juice after vacuum drying.

The color of food has a major impact on consumer choice and acceptance. This is due to the fact that the products chosen by consumers are visually appealing; therefore, it is important that the products obtained after the drying process retain their desired color [10].

The use of vacuum impregnation resulted in a decrease in the brightness index. In the case of onion juice, the decrease in L* for the fresh and dried material was insignificant, while a significant decrease (by 29.99%) was observed after vacuum drying. As expected, the use of beetroot juice as an impregnation solution resulted in a decrease in brightness of 18.90% for KaB, 14.52% for KaB_FD and 51.35% for KaB_VD. This is related to the very intense, dark color of beetroots [13].

Raw cauliflower showed the lowest color saturation value (C*) (11.50). The color saturation of the material after vacuum impregnation with onion juice was higher by 11.93%, and with beetroot juice by 30.22%. Of all the drying methods tested, the lowest saturation value was observed for CaB_VD, and the highest for CaC_VD. This may be due to the initial color of the cauliflower after the pretreatment, especially with beetroot juice (Figure 2). In the case of CaB_VD, a 39.71% decrease in saturation was recorded compared to Ca_VD. This may be related to the impregnation solution used and the higher drying temperature.

The highest increase in browning index (BI) was observed in fresh material after VI with the use of beetroot juice. In the case of fresh cauliflower after VI, an increase in BI of 187.82% was observed, while after the application of onion juice, the increase was 15.82%. Of the drying methods tested, a reduction in the browning index was observed in all tested material combinations in the FD-dried samples.

The total color difference is defined by ∆E, for which low values are favorable [37]. The total color change in the dried cauliflower compared to the fresh cauliflower was lowest for FD and highest for VD. Other researchers have also reported lower ∆E values for freeze-drying compared to other drying methods, e.g., *P. fortuneana fruits* [37]. In addition, much higher ∆E values were observed for cauliflower after VI with beetroot juice; these results are related to the greater difference in brightness (L*) and a* coordinate compared to fresh cauliflower that was not pretreated.

### 2.5. Texture Profile Analysis (TPA)

**Figure 3:**
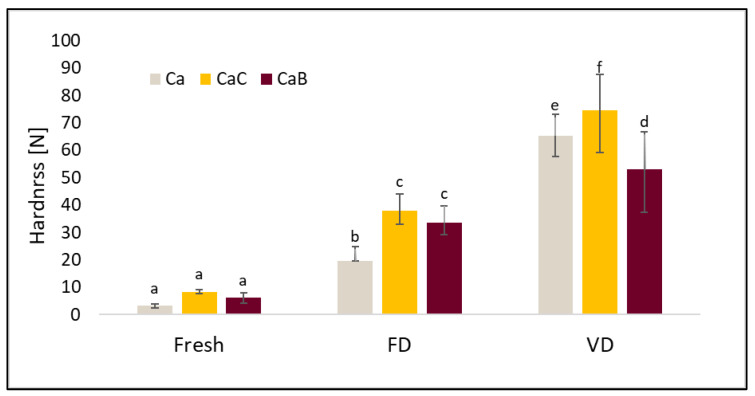
The effect of vacuum impregnation on the hardness of cauliflower (Ca), cauliflower after VI with onion juice (CaC), and cauliflower after VI with beetroot juice (CaB). FD—freeze-drying, VD—vacuum drying. The values marked with different lowercase letters indicate significant differences (*p* < 0.05).

**Figure 4:**
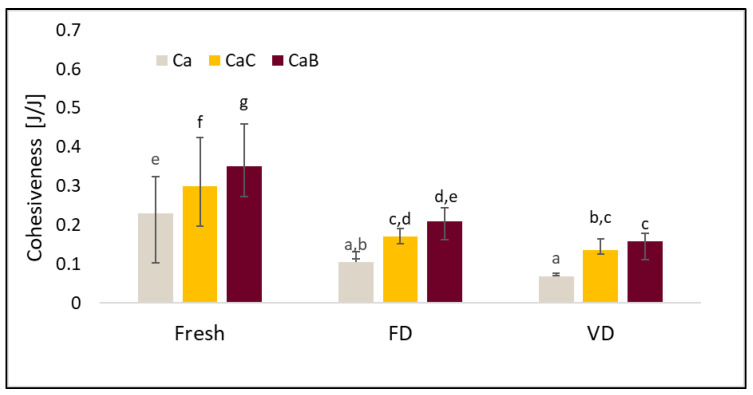
The effect of vacuum impregnation on the cohesiveness of cauliflower (Ca), cauliflower after VI with onion juice (CaC), and cauliflower after VI with beetroot juice (CaB). FD—freeze-drying, VD—vacuum drying. The values marked with different lowercase letters indicate significant differences (*p* < 0.05).

**Figure 5:**
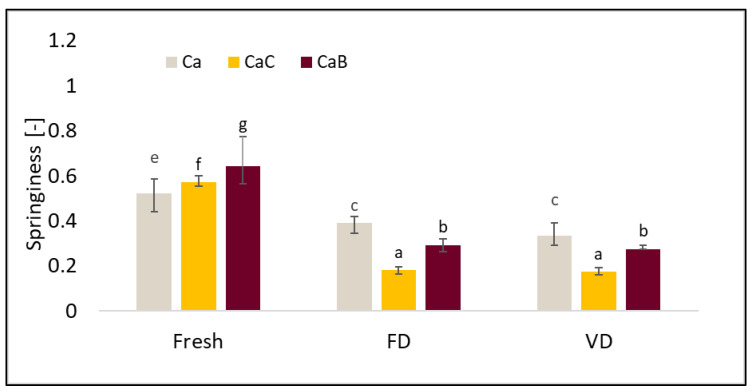
The effect of vacuum impregnation on the springiness of cauliflower (Ca), cauliflower after VI with onion juice (CaC), and cauliflower after VI with beetroot juice (CaB). FD—freeze-drying, VD—vacuum drying. The values marked with different lowercase letters indicate significant differences (*p* < 0.05).

**Figure 6:**
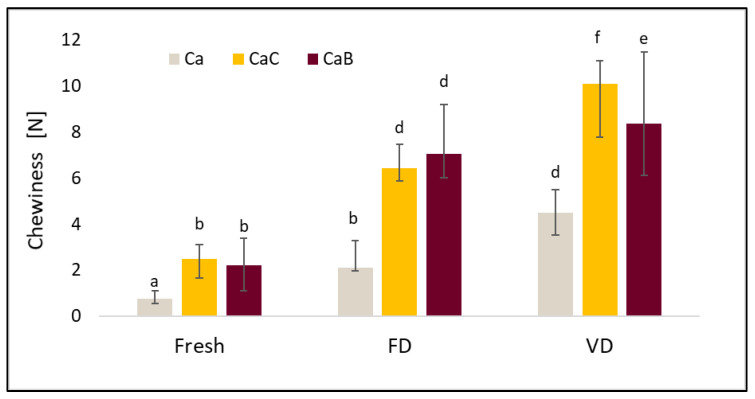
The effect of vacuum impregnation on the chewiness of cauliflower (Ca), cauliflower after VI with onion juice (CaC), and cauliflower after VI with beetroot juice (CaB). FD—freeze-drying, VD—vacuum drying. The values marked with different lowercase letters indicate significant differences (*p* < 0.05).

TPA data such as hardness, elasticity, cohesiveness, and ductility are presented in Figure 3, Figure 4, Figure 5 and Figure 6.

Figure 3 shows the average hardness values of fresh (Fresh), freeze-dried (FD) and vacuum-dried (VD) cauliflower. Fresh materials were characterized by the lowest hardness, and their firmness ranged from 3.25 N to 8.39 N depending on the vacuum impregnation variant. Of the drying methods tested, the lowest hardness values were recorded for freeze-drying. The dried samples after vacuum drying had higher values of the tested parameter by 231% for Ca, 96.8% for CaC, and 57.7% for CaB, respectively, compared to the freeze-drying method. The test results indicate that the use of the VI pretreatment increases the value of the parameter tested. In addition, the type of impregnation solution used had an impact on the results obtained. Among the different combinations of materials, the highest hardness values were recorded for cauliflower after treatment with onion juice. This may be related to the impregnation solution, which contained particles of crushed onion.

The cohesion of the cauliflower is shown in Figure 4. The highest values of this parameter were recorded for fresh cauliflower. In addition, an increase in cohesion was observed in the fresh and dried materials after vacuum impregnation. The value of the tested parameter was also influenced by the impregnation solution used (Figure 4). Other researchers testing the effect of vacuum impregnation observed an increase in cohesiveness in tilapia filets after using four different combinations of impregnation solutions [17].

The relationships concerning the elasticity of the material were similar to those noted for cohesiveness. The highest values of the tested parameter were noted for cauliflower not subjected to the drying process, and the values were as follows: 0.52 (Ca), 0.57 (CaC), and 0.64 (CaB). Both drying processes caused a decrease in the elasticity of the materials, with the lowest values of this parameter recorded for cauliflower after VD (Figure 5). It is worth noting that the use of VI increased the values of the tested parameter, both with onion juice and beetroot juice.

Figure 6 shows the results of the chewiness tests. The lowest values were recorded for the fresh, un-dried material. The use of freeze-drying allowed for better preservation of chewiness and a lower increase in this parameter compared to vacuum drying. Similarly, research conducted by Xu, H. et al. [37] showed that, of the many drying methods tested, materials after freeze-drying achieved the lowest chewiness values [37]. The authors reported values for chewing in the case of the sublimation method at a level of 688.33 N, while for materials hot-air-dried at 60 °C, the value of the studied parameter was 1927.62 N, and the result after vacuum drying at 60 °C was 1026.69 N [37].

## 3. Materials and Methods

### 3.1. Preparation of Sample

Good-quality cauliflowers were purchased at a local vegetable market (Wrocław, Poland). The vegetables were cut into pieces of uniform size (1.5 × 2.0 cm) and then washed with water. Before the tests, the vegetables were stored at −18 ± 1 °C. The experiments were carried out on samples that had been thawed and brought to room temperature.

### 3.2. Pretreatment Before Drying Process

The vacuum impregnation processes were carried out in a prototype installation, the diagram of which was presented in an earlier publication [16]. Freshly squeezed onion juice and organic red beetroot juice with an energy value of 155 kJ/37 kcal (Haus Rabenhorst, Unkel, Germany) were used for impregnation.

A vacuum impregnation process was used as pretreatment. VI was carried out according to the procedure described in our previous publication [14]. For each pretreatment, three independent vacuum impregnations were performed. After impregnation, the weight gain (WG) was calculated using the following equation, according to Tappi et al. (2022) [39]:

(1) $$WG=100\cdot \frac{m-m_{0}}{m_{0}}$$

where *m* is the mass of the impregnated sample and *m*_0_ is the initial mass.

The samples were prepared in 9 combinations (Table 6). After the vacuum impregnation process, the samples were tested for selected physical properties and an analysis of volatile compounds was carried out. The remaining samples were subjected to the drying process.

### 3.3. Drying

The drying of the cauliflower was carried out using two methods: the freeze-drying method (FD) and the vacuum drying method (VD). The freeze-drying process was carried out using a lyophilizer (Free-Zone 4.5 L, Labconco, Fort Scott, KS, USA) which used the contact heating method. Before drying, the samples were frozen and stored in an RL58GRGIH freezer (Samsung Electronics Polska sp. z.o.o., Wronki, Poland) for 24 h. Freeze-drying was carried out at a temperature of −50 °C with a pressure of 5 Pa in the chamber. The temperature of the heating plates was 22 °C. Vacuum drying was carried out in a V0101 dryer (Memmert, Schwabach, Germany) at a temperature of 60 °C and a pressure of 5 kPa. The drying time was 24 h.

### 3.4. VOC Extraction and Analysis

#### 3.4.1. Methods

The HS-SPME technique was used to extract volatile compounds. For this, 200 mg of dried material and 200 mg of homogenized fresh material were weighed and placed into 20 mL headspace glass vials. As an internal standard, 50 µL of 2-undecanone (Sigma-Aldrich, Steinheim, Germany) at a concentration of 0.1 mg/mL was added. In addition, 200 µL of distilled water was added into each vial. The extraction was performed with a 2 cm fiber (DVB/CAR/PDMS, Supelco, Bellefonte, PA, USA) which was pre-conditioned at 250 °C for 5 min. Meanwhile, the samples were incubated at 60 °C for 5 min. Thereafter, the extraction was carried out for 10 min at 60 °C, followed by a 3 min thermal desorption in the GC injection port at 250 °C.

Analyte separation and identification were carried out using a Shimadzu QP 2020 Plus (Shimadzu, Kyoto, Japan) equipped with a ZB-5Msi column (Phenomenex, Torrance, CA, USA) with dimensions of 30 m × 0.25 mm × 0.25 µm. The injector temperature was set to 250 °C, and helium was used as the carrier gas at a flow rate of 1.0 mL·min^−1^ with a linear velocity of 36.3 cm·s^−1^ and a split ratio of 10. The temperature program began at 50 °C, then increased to 130 °C at 3 °C·min^−1^, followed by 180 °C at 10 °C·min^−1^, and finally 280 °C at 20 °C·min^−1^. All analyses were conducted in triplicate.

#### 3.4.2. Identification

The identification of compounds was based on a comparison of the obtained mass spectra with the NIST 20 (National Institute of Standards and Technology) and FFNSC (Mass Spectra of Flavors and Fragrances of Natural and Synthetic Compounds) databases. The calculated linear retention indices (LRIs) were also compared using a retention index calculator, with values taken from NIST 20 and FFNSC. Additionally, the AMDIS (v. 2.73) and GCMS Postrun Analysis programs version 4 (Shimadzu, Kyoto, Japan) were employed for further analysis. The identification was further supported by the reference Identification of Essential Oil Components by Gas Chromatography/Mass Spectrometry (4.1 ed.) by Dr. Robert P. Adams, Professor at the Biology Department, Baylor University [40].

### 3.5. Physical Properties

The dry mass (DM) was measured using a dryer (Memmert, VO101, Schwabach, Germany). The samples were placed in a drying chamber at a reduced pressure (5 kPa), a temperature of 70 °C, and a drying time of 24 h. The measurements were taken in triplicate and the result was the average of the measurements.

The water activity (AW) was measured at a constant temperature of 25 °C using AquaLab 4TE ± 0.003 (AquaLab, Warsaw, Poland). The measurements were taken in quadruplicate.

The volumetric density was measured using the methodology of Tibiru et al. [41]. The measurement was performed in five repetitions.

The color coordinates of cauliflower when fresh, after the vacuum impregnation process, and dried, were evaluated using the Minolta Chroma Meter CR-200 (Minolta Corp., Osaka, Japan). Cauliflower has an uneven structure; therefore, in order to standardize the test results, the measurements were performed in eight repetitions and then the average was calculated. The color coordinates were plotted in the CIELab* colorimetric system, which included the following parameters: lightness (L*), where 100 means white and 0 means black; the a* parameter (negative values refer to green, positive values to red); and the b* parameter (negative values indicate blue, positive values indicate yellow). Using the three color parameters (L*, a*, b*), the color saturation (C*), the browning index (BI), and the total color change (∆E) were determined according to the literature [16].

The recorded texture properties included hardness, cohesiveness, springiness, and gumminess. Fresh samples, samples after vacuum impregnation with onion and beetroot juice, and dried samples were evaluated using an Instron 5544 testing machine (Instron Corporation, Canton, MA, USA). TPA was performed according to the methodology presented by Fi-giel and Tajner-Czopek [42]. The parameters were set as follows: detection speed of 1 mm/s, 50% deformation, and 5 N trigger force. All the tests were repeated six times, and the average was recorded.

### 3.6. Statistical Analysis

Statistical analyses were performed using Statistica version 13.3 (StatSoft, Kraków, Polska). One-way analysis of variance (ANOVA) using Duncan’s test was used to compare the mean values. Differences were considered to be significant at *p* < 0.05. The significant differences among the major VOCs were verified by hierarchical cluster analysis (HCA). Before the analysis, the used numerical data were standardized according to the software algorithm. The assumptions applied for HCA were as follows: Ward’s linkage, Euclidean distance, and the strict (33%) Sneath’s criterium.

## 4. Conclusions

This research has shown, for the first time, that the use of vacuum impregnation with onion and beetroot juice significantly affects the properties of white cauliflower. Twenty-two volatile compounds were identified for cauliflower, twenty-nine VOCs for cauliflower after vacuum impregnation with beetroot juice, and twenty-four VOCs for cauliflower after vacuum impregnation with onion juice. These results confirmed that the use of the vacuum impregnation process had a positive effect on the content of volatile compounds, and more VOCs were noted in the material after VI. The following volatile compounds were present in the highest amounts: 1-heptene, 2-methyl-(>60%), 2-ethylcyclobutanol (>4%), nona-3,5-dien-2-one (>1.8%), and two unidentified compounds, unknown 1 (probably an isomer of nona-3,5-dien-2-one (>1.8%)) and unknown 2 (probably a fatty acid) (>9.8%).

The use of the vacuum impregnation process reduced water activity and brightness. In addition, the material after VI showed an increased dry matter content, bulk density, color saturation, chewiness, elasticity, and cohesiveness. The use of beetroot juice resulted in a darker red color of the materials, an increase in the browning index, and a greater change in color.

It was observed that the cauliflower dried using the freeze-drying method had a lower water activity, higher dry matter content, lower density, increased brightness, smaller color difference, lower hardness, lower chewiness, higher cohesiveness, and higher elasticity. Therefore, this method is recommended for drying cauliflower. This study provides valuable information on the interaction between vacuum impregnation, impregnation solution, and drying agent temperature on the physicochemical and volatile properties of cauliflower. This research provides a solid foundation for the use of processed cauliflower in various food applications (snacks, soups, and salad additions).

## 5. Patents

Patent Poland, no. 421913. A vacuum impregnating machine and method for the initial processing of materials. Wrocław University of Environmental and Life Sciences, Wrocław, PL. Authors: Bogdan Stępień, Radosław Maślankowski, Leszek Rydzak, and Marta Pasławska.

## Figures and Tables

**Table 1:** HS-SPME arrow VOC profile for fresh cauliflower.

	Compounds	LRI Exp ^1^	LRI Lit ^2^	Match ^3^	Ca%	Ca SD ^4^	CaB%	CaB SD	CaC%	CaC SD
**1**	Unknown	781			nd ^5^	nd	nd	nd	11.33	1.95
**2**	Heptene <2-methyl-1->	784	784	89	63.54	6.06	72.86	7.86	61.55	1.18
**3**	Pentanethiol <4-methyl-2->	804	793	89	nd	nd	1.11	0.68	nd	nd
**4**	Hexanal	791	801	85	3.39	1.29	nd	nd	nd	nd
**5**	Ethylcyclobutanol<2->	831	828	90	7.56	4.01	4.86	3.62	4.62	1.77
**6**	Hexenal-<2E>	864	855	95	1.17	0.56	0.76	0.21	0.88	0.04
**7**	Hexanol <1>	875	867	93	nd	nd	nd	nd	0.16	0.01
**8**	Heptanal	901	906	90	0.20	0.11	0.14	0.10	0.09	0.04
**9**	Sorbaldehyde	909	914	94	nd	nd	nd	nd	0.17	0.08
**10**	Pinene <alpha->	932	933	96	0.55	0.51	0.64	0.50	0.27	0.08
**11**	Camphene	948	943	95	0.17	0.17	0.20	0.18	0.08	0.03
**12**	Heptenal <2E->	953	956	90	0.13	0.09	nd	nd	0.08	0.02
**13**	Benzaldehyde	958	960	98	1.46	0.83	0.72	0.50	0.83	0.26
**14**	Dimethyl trisulfide	966	964	90	nd	nd	nd	nd	0.67	0.65
**15**	Hexanoic acid	969	967	90	nd	nd	0.10	0.07	nd	nd
**16**	Sabinene	971	972	86	0.07	0.07	nd	nd	nd	nd
**17**	Pinene <beta->	977	978	90	0.18	0.19	0.23	0.21	nd	nd
**18**	Octene <2-methyl-6-methylene-2->	978	968	90	nd	nd	nd	nd	0.62	0.34
**19**	Myrcene	988	991	95	0.33	0.36	0.90	1.07	0.09	0.04
**20**	Pentyl furan <2->	994	988	91	nd	nd	0.24	0.07	nd	nd
**21**	Mentha-1(7),8-diene <meta->	1009	1001	85	nd	nd	0.71	0.30	nd	nd
**22**	Heptadienal <2,4-trans,trans->	1009	1013	88	1.71	0.80	nd	nd	0.80	0.16
**23**	Cymene <ortho->	1022	1024	92	0.18	0.15	0.21	0.20	nd	nd
**24**	Hexanol <2-ethyl->	1026	1029	92	nd	nd	nd	nd	0.24	0.01
**25**	Limonene	1028	1030	93	0.42	0.34	0.56	0.54	0.14	0.03
**26**	Eucalyptol	1031	1032	95	0.41	0.43	0.34	0.44	nd	nd
**27**	Oct-3-en-2-one	1035	1036	92	nd	nd	0.15	0.10	0.19	0.07
**28**	Decane <2-methyl->	1053	1051	90	nd	nd	0.06	0.05	nd	nd
**29**	Octenal <2->	1054	1059	93	nd	nd	0.07	0.04	0.06	0.02
**30**	Nona-3,5-dien-2-one	1065	1068	87	4.43	1.99	1.85	0.92	2.23	0.70
**31**	Unknown (probably isomer of previous compound)	1089			5.28	2.74	1.38	0.57	2.49	1.03
**32**	Undecane <n->	1099	1100	95	nd	nd	0.07	0.04	nd	nd
**33**	Nonanal	1102	1104	92	0.22	0.12	0.18	0.05	0.12	0.02
**34**	Thujone <beta->	1104	1118	90	0.08	0.09	0.09	0.09	nd	nd
**35**	Unknown (probably fatty acid)	1308			9.86	4.27	13.05	2.38	12.26	1.09

^1^ LRI exp—experimentally calculated LRI; ^2^ LRI lit—LRI available in library; ^3^ Mass spectra similarity match [%]; ^4^ standard deviation; ^5^ nd—not detected; Ca—cauliflower; CaC—cauliflower with onion juice; CaB—cauliflower with beetroot juice.

**Table 2:** HS-SPME arrow VOC profile for dried cauliflower.

	Compounds	LRI Exp ^1^	LRI Lit ^2^	Match ^3^	Ca FD%	CaB FD%	CaC FD%	Ca VD%	CaB VD%	CaC VD%
**1**	Butenolide	805	807	88	19.74 ± 2.62	nd ^4^	nd	2.16 ± 0.92	nd	nd
**2**	Formate <pentyl->	814	823	91	1.42 ± 0.44	nd	nd	3.55 ± 0.47	nd	nd
**3**	Ethylcyclobutanol<2->	830	828	89	12.44 ± 1.58	3.43 ± 0.53	3.55 ± 0.27	12.94 ± 0.39	3.51 ± 0.86	11.00 ± 1.71
**4**	Isovaleric acid	850	842	86	Nd	0.58 ± 0.20	0.77 ± 0.16	nd	1.66 ± 0.66	5.22 ± 1.18
**5**	Unknown	856			Nd	nd	0.38 ± 0.09	nd	nd ±0.04	2.32 ± 0.47
**6**	Hexenal <2E>	863	850	95	1.16 ± 0.25	0.59 ± 0.04	0.58 ± 0.03	1.11 ± 0.16	0.29 ± 0.00	1.09 ± 0.04
**7**	Isothiocyanate <2-propenyl->	883	880	93	nd	0.42 ± 0.08	nd	nd	nd	nd
**8**	Heptanal	906	900	95	0.36 ± 0.09	0.12 ± 0.01	0.12 ± 0.00	0.32 ± 0.07	0.16 ± 0.03	0.27 ± 0.03
**9**	Pyrazine <2,5-dimethyl->	910	912	90	1.05 ± 0.38	nd	0.43 ± 0.02	0.82 ± 0.15	nd	1.13 ± 0.32
**10**	Hexanoate <methyl->	920	922	94	0.33 ± 0.04	nd	nd	0.07 ± 0.01	nd	nd
**11**	Pinene <alpha->	932	933	98	nd	0.79 ± 0.01	0.84 ± 0.06	nd	0.68 ± 0.13	0.38 ± 0.06
**12**	Camphene	948	953	97	0.37 ± 0.22	1.27 ± 0.02	1.28 ± 0.05	0.42 ± 0.22	1.06 ± 0.22	0.58 ± 0.08
**13**	Heptenal <2E>	952	956	91	0.19 ± 0.02	nd	nd	0.17 ± 0.02	nd	nd
**14**	Benzaldehyde	957	960	98	4.77 ± 1.29	1.13 ± 0.14	1.75 ± 0.20	17.38 ± 4.95	6.66 ± 1.14	9.11 ± 0.47
**15**	Trisulfide <dimethyl->	965	969	85	0.83 ± 0.46	nd	0.49 ± 0.08	0.52 ± 0.04	nd	1.95 ± 0.62
**16**	Hexanoic acid	971	974	95	nd	nd	0.78 ± 0.15	nd	nd	1.30 ± 0.18
**17**	Pinene <beta->	976	978	90	nd	0.33 ± 0.25	0.34 ± 0.02	nd	0.27 ± 0.10	0.14 ± 0.03
**18**	Caproic acid	971	979	92	4.04 ± 2.35	0.53 ± 0.00	nd	1.34 ± 0.40	0.23 ± 0.04	nd
**19**	Oct-1-en-3-ol	977	978	87	0.87 ± 0.13	nd	nd	1.05 ± 0.12	nd	nd
**20**	Unknown	982			1.19 ± 0.36	nd	nd	0.78 ± 0.15	nd	nd
**21**	Hept-5-en-2-one <6-methyl->	981	986	85	nd	2.15 ± 0.13	1.86 ± 0.06	nd	1.91 ± 0.62	1.47 ± 0.26
**22**	Myrcene	988	991	98	15.24 ± 11.93	62.09 ± 0.59	62.10 ± 1.69	13.98 ± 8.30	54.61 ± 5.27	22.79 ± 3.79
**23**	Pyrazine <2-ethyl-, 6-methyl->	995	994	90	1.06 ± 0.40	0.59 ± 0.12	0.54 ± 0.07	0.80 ± 0.15	1.96 ± 0.45	1.35 ± 0.31
**24**	Decane	1000	1000	90	nd	0.55 ± 0.04	0.69 ± 0.02	nd	0.10 ± 0.04	0.55 ± 0.06
**25**	Mentha-1(7),8-diene <p->	1003	1004	97	nd	3.28 ± 0.08	3.29 ± 0.08	nd	2.72 ± 0.39	1.21 ± 0.19
**26**	Heptadienal <2,4-trans,trans->	1009	1013	90	1.03 ± 0.21	nd	0.37 ± 0.07	1.67 ± 0.19	nd	1.67 ± 0.19
**27**	Cymene <para->	1022	1025	96	nd	1.81 ± 0.10	1.70 ± 0.06	nd	1.73 ± 0.18	0.71 ± 0.11
**28**	Hexanol <2-ethyl->	1026	1028	90	1.40 ± 0.37	0.49 ± 0.04	0.76 ± 0.02	0.26 ± 0.08	0.32 ± 0.04	0.39 ± 0.02
**29**	Limonene	1027	1030	90	nd	0.39 ± 0.02	0.37 ± 0.02	nd	0.35 ± 0.02	0.27 ± 0.00
**30**	3-Octen-2-one	1034	1047	93	0.61 ± 0.12	nd	nd	0.51 ± 0.04	nd	nd
**31**	Nona-3,5-dien-2-one	1065	1068	86	5.24 ± 1.18	2.24 ± 0.08	2.11 ± 0.26	10.24 ± 0.80	0.53 ± 0.18	7.89 ± 0.76
**32**	Unknown (probably isomer of previous compound)	1090			6.62 ± 1.74	1.82 ± 0.01	2.07 ± 0.04	7.01 ± 0.69	0.13 ± 0.02	6.05 ± 0.88
**33**	Nonanal	1102	1104	93	nd	nd	0.22 ± 0.01	nd	nd	0.48 ± 0.01
**34**	Geranial	1160	1174	86	nd	0.19 ± 0.01	0.14 ± 0.01	nd	0.13 ± 0.05	0.06 ± 0.03
**35**	Citral	1183	1174	90	nd	0.19 ± 0.03	0.13 ± 0.01	nd	0.12 ± 0.03	0.03 ± 0.01
**36**	Dodecane	1199	1200	96	5.29 ± 2.28	3.33 ± 0.52	3.93 ± 0.29	1.48 ± 0.45	0.19 ± 0.04	1.09 ± 0.21
**37**	Unknown (probably fatty acid)	1308			12.40 ± 2.53	9.85 ± 0.64	6.45 ± 0.72	22.98 ± 2.27	20.79 ± 3.47	18.83 ± 2.26
**38**	Tetradecane <n->	1400	1400	95	3.54 ± 1.17	1.86 ± 0.11	1.97 ± 0.05	1.12 ± 0.22	0.15 ± 0.05	0.82 ± 0.08

^1^ LRI exp—experimentally calculated LRI; ^2^ LRI lit—LRI available in library; ^3^ Mass spectra similarity match [%]; ^4^ nd—not detected; Ca—cauliflower; CaC—cauliflower with onion juice; CaB—cauliflower with beetroot juice; FD—freeze-drying; VD—vacuum drying.

**Table 3:** Physicochemical parameters of vacuum-impregnated samples fresh and immediately after vacuum impregnation (VI) treatment. WG: weight gain.

Material	WG	°Bx
CaC	4.03 ± 1.43	11.5 ± 0.08
CaB	8.90 ± 1.11	7.5 ± 0.12

Ca: cauliflower; CaC: cauliflower with onion juice; CaB: cauliflower with beetroot juice.

**Table 4:** Water activity (AW), dry mass (DM), and bulk density.

Method	Water Activity [-]	Dry Mass [%]	Bulk Density [kg/m^3^]
Ca	0.970 ± 0.006 ^d^	8.91 ± 0.24	275.44 ± 8.20 ^d^
Ca_FD	0.145 ± 0.011 ^a^	98.64 ± 0.89	30.71 ± 2.31 ^a^
Ca_VD	0.294 ± 0.016 ^b^	97.94 ± 0.57	122.14 ± 7.35 ^b^
CaC	0.978 ± 0.002 ^e^	9.22 ± 0.16	365.94 ± 14.49 ^d^
CaC_FD	0.173 ± 0.006 ^a^	96.09 ± 3.22	36.69 ± 4.32 ^a^
CaC_VD	0.369 ± 0.013 ^c^	95.39 ± 2.90	160.11 ± 1.74 ^c^
CaB	0.982 ± 0.001 ^d^	9.88 ± 0.15	368.23 ± 13.55 ^d^
CaB_FD	0.257 ± 0.016 ^b^	96.44 ± 0.70	35.78 ± 1.70 ^a^
CaB_VD	0.328 ± 0.034 ^c^	95.74 ± 0.72	160.99 ± 3.89 ^c^

The values (mean of three replications) ± standard deviation followed by different letters (^a–e^ are different (*p* ≤ 0.05) according to Duncan’s test. Ca: cauliflower; CaC: cauliflower with onion juice; CaB: cauliflower with beetroot juice; FD: freeze-drying; VD: vacuum drying.

**Table 5:** Color parameters of raw and dried cauliflower: L*—lightness; a*—(+) redness/(−) greenness; b* —(+) yellowing; BI—browning index; C*—saturation; ΔE—total color of vegetables.

Method	L*	a*	b*	C*	BI	∆E
Ca	73.10 ± 3.20 ^c^	−0.65 ± 0.19 ^f^	11.53 ± 1.79 ^e^	11.50 ± 1.81	16.27 ± 2.70	-
Ca_FD	81.45 ± 3.96 ^a^	1.80 ± 0.46 ^d^	16.55 ± 1.68 ^b^	16.60 ± 1.68	26.30 ± 3.28	9.87
Ca_VD	79.84 ± 6.25 ^b^	0.40 ± 0.21 ^e^	20.61 ± 1.93 ^a^	20.62 ± 1.93	29.77 ± 2.22	11.31
CaC	71.33 ± 4.39 ^c^	−0.87 ± 0.45 ^f^	12.91 ± 2.73 ^d^	12.87 ± 2.75	18.84 ± 4.60	2.48
CaC_FD	72.54 ± 4.60 ^c^	2.12 ± 1.45 ^d^	20.46 ± 3.96 ^a^	20.54 ± 3.98	35.30 ± 9.52	9.18
CaC_VD	51.17 ± 3.70 ^e^	11.98 ± 1.14 ^b^	20.78 ± 2.02 ^a^	21.07 ± 1.99	68.96 ± 6.87	26.65
CaB	59.28 ± 2.52 ^e^	15.67 ± 2.52 ^a^	14.44 ± 1.85 ^c^	14.98 ± 1.81	46.83 ± 5.33	20.95
CaB_FD	62.48 ± 2.66 ^d^	15.44 ± 0.47 ^a^	19.01 ± 1.38 ^a^	19.41 ± 1.36	54.06 ± 3.80	20.03
CaB_VD	35.56 ± 3.67 ^f^	9.58 ± 1.80 ^c^	12.03 ± 1.36 ^d^	12.43 ± 1.27	61.70 ± 9.17	38.70

The values (mean of three replications) ± standard deviation followed by different letters (a–f) are different (*p* ≤ 0.05) according to Duncan’s test. Ca: cauliflower; CaC: cauliflower with onion juice; CaB: cauliflower with beetroot juice; FD: freeze-drying; VD: vacuum drying.

**Table 6:** The explanation of the samples coding.

Code	Material	Type of Drying
Ca	Cauliflower	-
Ca_FD	Cauliflower	freeze-drying
Ca_VD	Cauliflower	vacuum drying
CaC	Cauliflower with onion juice	-
CaC_FD	Cauliflower with onion juice	freeze-drying
CaC_VD	Cauliflower with onion juice	vacuum drying
CaB	Cauliflower with beetroot juice	-
CaB_FD	Cauliflower with beetroot juice	freeze-drying
CaB_VD	Cauliflower with beetroot juice	vacuum drying

## Data Availability

Data are contained within the article.
